# Biodegradation of poly (ε-caprolactone) (PCL) film and foam plastic by *Pseudozyma japonica* sp. nov., a novel cutinolytic ustilaginomycetous yeast species

**DOI:** 10.1007/s13205-013-0182-9

**Published:** 2013-11-13

**Authors:** Fatma F. Abdel-Motaal, Magdi A. El-Sayed, Soad A. El-Zayat, Shin-ichi Ito

**Affiliations:** 1 Department of Botany, Faculty of Science, Aswan University, Aswân, 81528 Egypt; 2 Department of Biological and Environmental Sciences, Faculty of Agriculture, Yamaguchi University, Yamaguchi, 753-8515 Japan

**Keywords:** Plastic degradation, PCL degradation, Foam degradation, Yeast, *Pseudozyma*

## Abstract

Aliphatic polyesters poly (ε-caprolactone) (PCL) and foam plastic have been shown to be biodegradable by microorganisms, which possess cutinolytic enzymes. *Pseudozyma japonica*-Y7-09, showed both high growth and enzyme activity on Yeast malt (YM) medium fed with PCL film than on YM medium. The hydrolytic enzyme activity of the culture on *p*-nitrophenyl butyrate indicated the occurrence of cutinase enzyme. This activity was confirmed by the degradation of PCL film which reached to the maximum (93.33 %) at 15 days and the degradation of foam plastic which reached 43.2 % at 30 days. These results suggest that the extracellular cutinase enzyme of *Pseudozyma japonica*-Y7-09 may be useful for the biological degradation of plastic wastes.

## Introduction

Plastics become the essential ingredients to provide a quality due to their versatility. These are now rival metals in breadth of use and in severity of applications because of their flexibility, toughness, excellent barrier and physical properties and ease of fabrication (Seymour 1971; Andrady et al. 1998; Meenakshi et al. 2002; Fang and Fowler 2003; Orhan et al. 2004). The growing amount of plastic waste is generating more and more environmental problems worldwide. To overcome this problem, the biodegradation of plastics has been subjected to extensive studies during the past three decades. Biodegradation of plastics is seen by many as a promising solution to this problem as it is environmentally-friendly. Some types of plastics have been shown to be biodegradable by microorganisms which produce enzymes. Those biodegradable plastics such as aliphatic polyesters (polycaprolactone, PCL) which are used mainly in thermoplastic polyurethanes, resins for surface coatings, adhesives for shoes and synthetic leather and fabrics, also serve to make stiffeners for shoes and orthopaedic splints, and fully biodegradable compostable bags, sutures and fibres. The chemical structure of a PCL trimer is similar to two cutin monomers, which are inducer for cutinase activity. This knowledge was helpful to find microorganisms which are able to degrade PCL (Premraj and Doble 2005). Although numerous studies (El-Shafei et al. 1998; Howard 2003; Nishida and Tokiwa 1993; Tokiwa et al. 2009) have been reported on the biodegradation of different types of plastics, the published literature on the biodegradation of plastic foams appears to be scarce. Foam biodegradation is carried out by the enzymes associated with some microorganisms like bacteria and fungi (Gautam et al. 2007).

Recently, a special focus has been given to the endophytic microorganisms that live inside the plant tissue without causing any immediate, overt effects (Hirsch and Braun 1992). Endophytes are known to produce bioactive natural products, which offer an enormous potential for exploitation for medicinal, agricultural and industrial uses (Tan and Zou 2001; Zhang et al. 2006). Special concerns are given to endophytic yeasts isolated from different plants (Larran et al. 2001, 2002; Cao et al. 2002; Tian et al. 2004; Nassar et al. 2005; Xin et al. 2009). Among them, smut yeast-like fungi of the genus *Pseudozyma* have attracted considerable attention as producers of enzymes (Pandey et al. 1999). Seo et al. (2007) reported that *Pseudozyma jejuensis* is able to degrade plastics waste. However, very few information is available so far about the biochemical activity of endophytic *Pseudozyma* spp.

Although PCLs have been shown to be degraded by enzymes secreted by a number of bacteria (Benedict et al. 1983), little studies have been done using yeast. A new yeast strain isolated recently from the medicinal plant *Hyoscyamus muticus* in our laboratory was identified as a novel species of the genus *Pseudozyma japonica*-Y7-09, (Abdel-Motaal et al. 2009). Evaluation of the PCL and foam plastic biodegradation by this new species was the objective of this study.

## Materials and methods

### Fungal strain and media

The yeast *strain Pseudozyma japonica*-Y7-09, from *Hyoscyamus muticus* (Egyptian henbane) was isolated as a new species of the genus *Pseudozyma*.

### Yeast culture and PCL films, foam plastics degradation assay

The *Pseudozyma* strain was grown on the yeast malt agar (YMA) (glucose 1.0 % w/v, peptone 0.5 % w/v, yeast extract 0.3 % w/v, malt extract 0.3 % w/v, agar 2.0 % w/v), and the plates were incubated for 2 days at 25 °C. A single colony of yeast species was inoculated into 5 ml of YM broth and cultured at 30 °C. After 24 h, 1 ml of culture broth was inoculated into 1,000-ml flask contain 50 ml YM broth and incubated at 30 °C with shaking at 200 rpm over night. Sterilized dry PCL film of known weight was added to each culture in addition to positive control (YM broth culture of each *Pseudozyma* species without PCL film) and negative control (YM broth with PCL film only). Flasks were incubated at 30 °C with shaking at 200 rpm for 15 days. Three replicas from each flask have been done considering the different time intervals (3, 5, 8, 12 and 15 days) to study the degradation behaviour. Under the same conditions, foam plastics were incubated in yeast culture but for longer time (30 days). The PCL films and foams were taken out carefully in different intervals time, washed thoroughly by double distilled water to remove any media components or yeast cells if present on the their surface and were completely vacuum dried at 30 °C over night. The percentage of weight loss was recorded.

### Culture turbidity

Growth rate of yeast culture incubated with and without PCL films and foams was measured and assayed according to the culture turbidity (OD_600nm_).

### pH values

Effect of variable pH values on the growth rate, protein concentrations and enzyme activity have been studied.

### Preparation of polycaprolactone (PCL) film and market’s foam plastic

Poly (ε-caprolactone) (PCL) with molecular weight 70,000–100,000 Da was purchased from Sigma-Aldrich Chemical Co. PCL film was prepared in small glass petri dishes (3 cm) by dissolving polymer in acetone and left for air drying. The prepared PCL films and the 1 cm quadrate cutting foams (polystyrene with molecular weight 100,000–400,000 Da) were weighed and sterilized in 80 % ethanol for 2 min, well dried and then kept under UV lamp for 30 min and fed into the pre-grown yeast cultures.

### Cutinolytic enzyme assays

Spectrophotometric assay was used to determine the cutinase activity with *p*-nitrophenyl butyrate (PNB; Sigma, ST Louis, MO, USA) and *p*-nitrophenyl palmitate (PNP; Sigma, ST Louis, MO, USA) as substrates (Kim et al. 2003).

96-well microplate was used for the hydrolysis reactions at 30 °C for 4 min. Each well contained 200 μl of an enzyme/substrate solution (solution A), comprising 106.7 μl of phosphate buffer (0.1 M, pH 8.0), 13.3 μl of Triton X-100 (4 g l^−1^), 13.3 μl of cell-free culture broth and 66.7 μl of substrate (PNB or PNP) solution. The PNB and PNP solutions were prepared with variable concentrations ranging from 0 to 1,000 mg l^−1^. The initial velocity [i.e. the initial maximum rate of change in absorbance (∆OD_405nm_ s^−1^)] was measured using a Molecular Devices EMax 96-well microplate reader (MDS Analytical Technologies, Toronto, Canada), and the enzyme-free solution A was used as a blank. As the eight wells in each of the 12 columns of the 96-well microplate contained an equal content of enzyme and substrate, the eight initial velocities measured for each column were used to calculate an average initial velocity for a specific reaction condition, resulting in an SD <5 %.

### Protein assay

Protein concentrations were determined according to Bradford method using Bio-Rad reagent (Bradford 1976).

## Results and discussion

### Condition favouring the degradation of polycaprolactone

The rate of degradation of biodegradable plastics is controlled not only by their chemical structure but also by environmental conditions such as temperature (Kitamoto et al. 2011). The yeast *strain Pseudozyma japonica*-Y7-09 showed the best growth rate and degradation percentage of polycaprolactone (PCL) and foam plastics in yeast malt broth media at 30 °C and at pH 6 with shacking at 200 rpm. Maximal activity of the PNB and PNP was found in the same conditions.

### Enzyme activity of *Pseudozyma japonica*-Y7-09

**Fig. 1:**
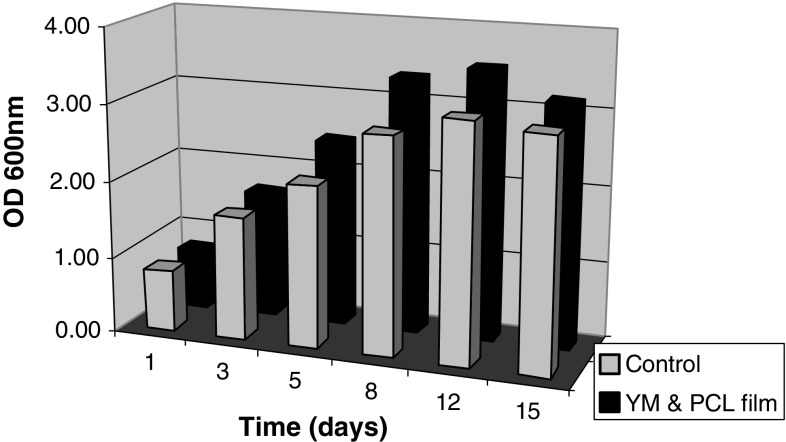
Growth measured by culture turbidity (OD_600nm_) in control (culture in YM) and culture in YM fed with PCL film (YM and PCL film)

**Fig. 2:**
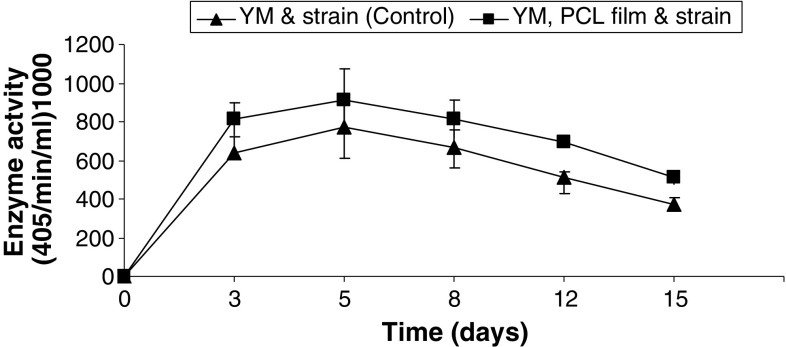
Activity of enzyme secreted by *Pseudozyma japonica*-Y7-09 in yeast malt culture fed and that not fed with PCL film

*Pseudozyma japonica*-Y7-09 showed higher growth on YM medium fed with PCL film than on YM medium (Fig. 1). The substrate-specific hydrolysis and high accessibility to PNB are intrinsic properties of cutinase that have never been observed for hydrolysis by lipases and esterases (Kim et al. 2003). Isolation of natural cutinase from variable microbial sources would be easy using this distinct characteristic. The hydrolytic activities of cell-free supernatant from *Pseudozyma japonica*-Y7-09 culture broth on two*p*-nitrophenyl esters (PNB and PNP) were estimated for a range of substrate concentrations on time courses. The hydrolytic activities are defined as the initial maximum rate of hydrolysis at each substrate concentration as described previously (Kim et al. 2003; Seo et al. 2007). On the time course, the hydrolytic enzyme activity of the culture on PNB rapidly increased after 3 days and the maximum activity was at day 5. The initial maximum rate of PNB hydrolysis in YM and PCL medium was higher than that in YM medium (Fig. 2), whereas the hydrolytic activity on PNP was very low with the same range of substrate concentrations, even in YM and PCL medium. Therefore, it is likely that the cutinase production by *Pseudozyma japonica*-Y7-09 was induced by PCL. These results are similar to those obtained by Kim et al. 2003, Seo et al. 2007 using *Pseudozyma jejuensis OL71*.

**Fig. 3:**
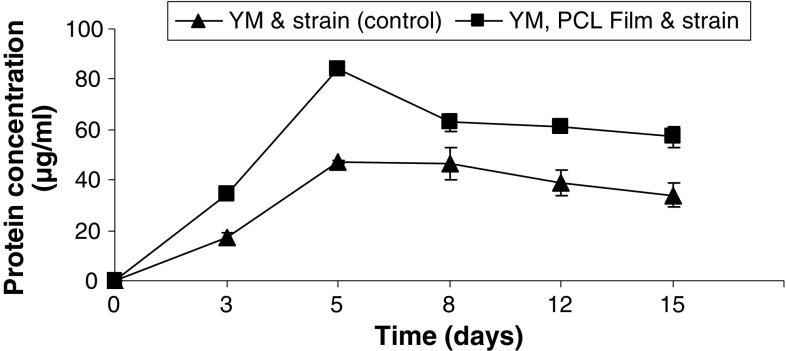
Total protein concentration secreted by *Pseudozyma japonica*-Y7-09 in yeast malt culture fed and that not fed with PCL film

During growth of *Pseudozyma japonica*-Y7-09 culture broth, the protein concentration rapidly increased after 3 days with a maximum concentration at 5 days similar to enzyme activity. Protein concentration recorded higher concentration in PCL feed YM medium than that in PCL free YM medium (Fig. 3).

### PCL degradation by *Pseudozyma japonica*-Y7-09

PCL dimers and trimers, products of enzymatic PCL degradation, are structurally similar to cutin degradation products that are known to be inducers of cutinase activity. This implies that oligomers of PCL may be natural inducers and substrates of cutinase (Nishida and Tokiwa 1993).

**Fig. 4:**
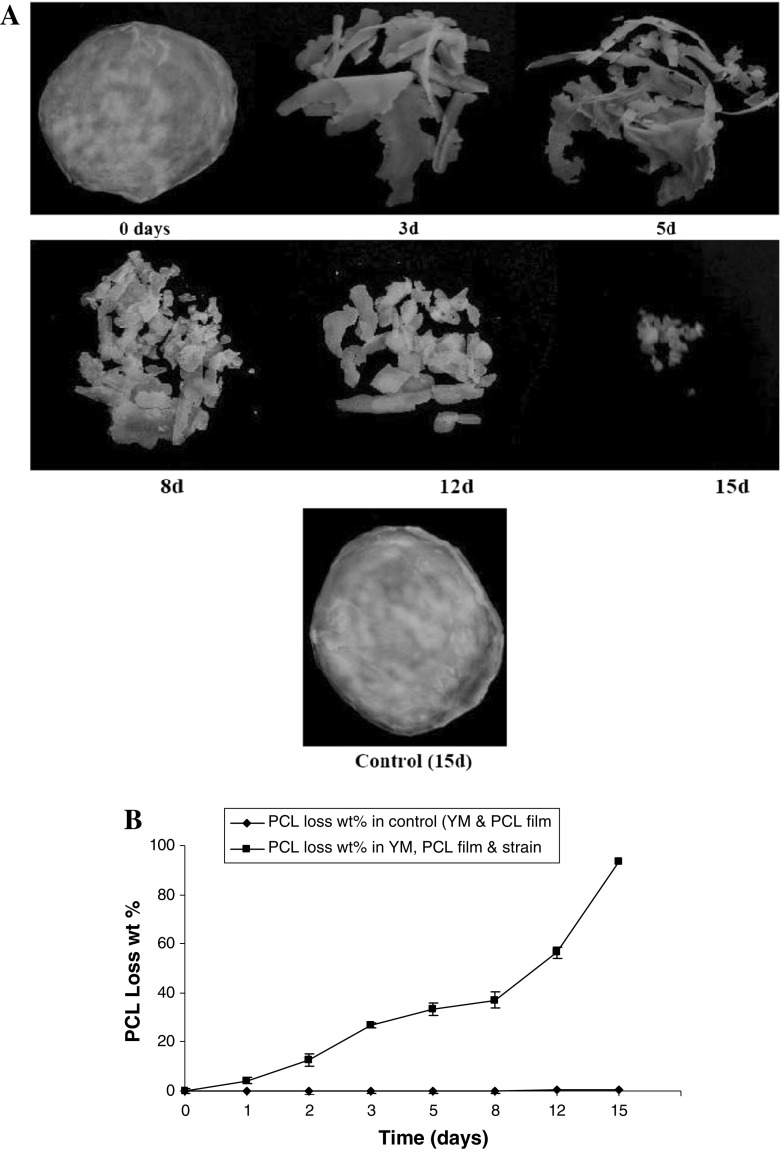
**a** Degradation of PCL film after 0, 3, 5, 8, 12 and 15 days incubation time by *Pseudozyma japonica*-Y7-09 comparing with control. **b** Weight loss percentage of PCL film after 0, 3, 5, 8, 12 and 15 days incubation time by *Pseudozyma japonica*-Y7-09 comparing with control

**Fig. 5:**
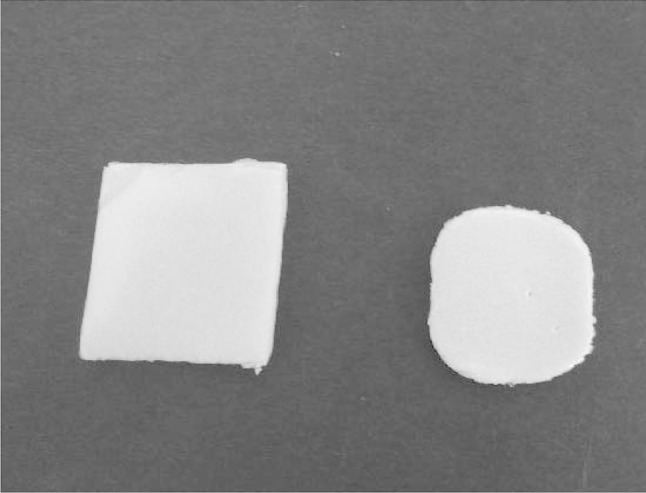
Degradation of foam plastic by *Pseudozyma japonica*-Y7-09 incubated in yeast malt media for 30 days

In the present study, PCL degradation was achieved in the conditions favouring the yeast strain’s growth in the YM medium in Erlenmeyer flasks as described above for 15 days. The weight loss of the degraded film was recorded and compared as a function of time. Under those conditions, partial degradation of PCL film (26.88 %) was recorded at the third day of incubation. That degradation reached to the maximum (93.33 %) at the 15 days (Figs. 4, 5). Weight loss was negligible in the same medium but free yeast cells (Figs. 4, 5). In an early study (Oda et al. 1995) isolated five strains of filamentous fungi of which *Paecilomyceslilacinus* was able to degrade 10 % of PCL after 10 days incubation. Xu et al. (2007) investigated the ability of several stock bacterial strains to degrade PCL. After 30 days of incubation, the weight loss of PCL film by the strain *Lysinibacillus* sp. 70,038 was 9 % while other strains showed less weight loss degradation.

### Foam degradation by *Pseudozyma japonica*-Y7-09

During the growth of *Pseudozyma japonica*-Y7-09 in YM medium fed with foam disc for 30 days, the extracellular culture broth secretion exhibited an ability to degrade the foam disc. The loss weight percentage of the foam disc within 30 days incubation time was 43.2 % (Fig. 5). This ability was slower as compared to PCL degradation but it is promising for the biodegradation of foam since foams are considered to be highly resistant to biodegradation when compared with other types of synthetics plastics (Gautam et al. 2007). An increase in molecular weight results in a decline of polymer degradability by microorganisms. High molecular weights result in a sharp decrease in solubility making polymers unfavourable for microbial attack due to difficulties of the substrate to be assimilated through the cellular membrane and then further degraded by cellular enzymes. The breakdown of large polymers to carbon dioxide (mineralization) requires several different organisms, with one breaking down the polymer into its constituent monomers, one able to use the monomers and excreting simpler waste compounds as by-products and one able to use the excreted wastes. Our yeast strain is capable of being one in a potential series of microbial strains used for degradation of plastic foam.

## Conclusions

*Pseudozyma japonica*-Y7-09 as a new yeast species exhibited high ability to degrade PCL and foam plastic. The hydrolytic activities of cell-free supernatant of *Pseudozyma japonica*-Y7-09 culture broth on two*p*-nitrophenyl esters (PNB and PNP) and its high accessibility to PNB are intrinsic properties of cutinase production. Further detailed studies of the enzymes produced by *Pseudozyma japonica*-Y7-09 to degrade other types of plastics are currently being undertaken, and the results of these will be important for the development of new technological processes for the biodegradation of plastic pollutants.
